# Paradoxical Relationship between Mn Superoxide Dismutase Deficiency and Radiation-Induced Cognitive Defects

**DOI:** 10.1371/journal.pone.0049367

**Published:** 2012-11-08

**Authors:** Rikki Corniola, Yani Zou, David Leu, John R. Fike, Ting-Ting Huang

**Affiliations:** 1 Department of Neurology and Neurological Sciences, Stanford University School of Medicine, Stanford, California, United States of America; 2 Palo Alto Institute for Research and Education, Palo Alto, California, United States of America; 3 Departments of Neurosurgery and Radiation Oncology, University of California San Francisco, San Francisco, California, United States of America; 4 Geriatric Research, Education, and Clinical Center (GRECC), VA Palo Alto Health Care System, Palo Alto, California, United States of America; University of Pecs Medical School, Hungary

## Abstract

Radiation therapy of the CNS, even at low doses, can lead to deficits in neurocognitive functions. Reduction in hippocampal neurogenesis is usually, but not always, associated with cognitive deficits resulting from radiation therapy. Generation of reactive oxygen species is considered the main cause of radiation-induced tissue injuries, and elevated levels of oxidative stress persist long after the initial cranial irradiation. Consequently, mutant mice with reduced levels of the mitochondrial antioxidant enzyme, Mn superoxide dismutase (MnSOD or Sod2), are expected to be more sensitive to radiation-induced changes in hippocampal neurogenesis and the related functions. In this study, we showed that MnSOD deficiency led to reduced generation of immature neurons in *Sod2*−/+ mice even though progenitor cell proliferation was not affected. Compared to irradiated *Sod2*+/+ mice, which showed cognitive defects and reduced differentiation of newborn cells towards the neuronal lineage, irradiated *Sod2*−/+ mice showed normal hippocampal-dependent cognitive functions and normal differentiation pattern for newborn neurons and astroglia. However, we also observed a disproportional decrease in newborn neurons in irradiated *Sod2*−/+ following behavioral studies, suggesting that MnSOD deficiency may render newborn neurons more sensitive to stress from behavioral trainings following cranial irradiation. A positive correlation between normal cognitive functions and normal dendritic spine densities in dentate granule cells was observed. The data suggest that maintenance of synaptic connections, via maintenance of dendritic spines, may be important for normal cognitive functions following cranial irradiation.

## Introduction

Radiation therapy is commonly used in the treatment of malignant brain tumors, and although effective, the dose that can be administered safely is limited due to potential injury to normal tissues [1], [2], [3], [4]. Radiation injury to the brain can involve multiple regions and a variety of cell/tissue types, leading to variable degrees of motor and cognitive dysfunctions [4]. The extent of injury is also influenced by a large number of physical and biological factors [2], [3]. Although there are considerable data available describing the various morphological outcomes in tissues following high and low radiation doses, the pathogenesis of these changes remains unclear.

Studies with experimental animals and humans suggest that the production of new neurons, (i.e. neurogenesis) continues in limited brain regions throughout the entire adult life [5], [6], [7]. Notably, adult neurogenesis occurs in the subgranular zone (SGZ) of hippocampal dentate gyrus and the process is important for hippocampal-dependent learning and memory [8], [9], [10]. However, hippocampal neurogenesis is exquisitely sensitive to irradiation and other stressors [11], [12]. Consequently, cranial irradiation therapy can have a strong negative impact on hippocampal neurogenesis and its associated functions of learning and memory.

The generation of reactive oxygen species (ROS) is considered a main cause of radiation-induced tissue damage [13]. Ionizing irradiation not only results in the acute generation of short-lived reactive oxygen species (ROS), it also results in a persistent state of oxidative stress that extends up to several months or even years after irradiation [14], [15], [16]. Altered levels of ROS are capable of influencing neuronal stem cell proliferation and differentiation [17], [18], [19], [20], [21] as well as synaptic plasticity and long-term potentiation [22], [23], [24]. Consequently, irradiation-induced alterations in hippocampal neurogenesis and synaptic plasticity are strongly implicated in cognitive impairments [2], [3], [4]. Unfortunately, there are no known effective treatments available that will help prevent the losses in adult neurogenesis and cognitive declines seen post-irradiation. Therefore, it is imperative to understand the molecular mechanisms of how oxidative stress regulates hippocampal neurogenesis and synaptic plasticity and, ultimately, learning and memory behaviors in response to radiation therapy.

Research is now starting to focus on manipulating the innate response mechanisms in neuronal precursor cells that respond to the effects of ROS. These mechanisms include the antioxidant enzyme superoxide dismutase (SOD). There are three genetically and geographically distinct isoforms of SOD, and all of them contribute to the conversion of superoxide anions to hydrogen peroxide. Hydrogen peroxide can then be catalyzed by peroxidases. Mn superoxide dismutase (MnSOD or Sod2) is one of the SOD isoforms and is located in the matrix of mitochondria. High levels of MnSOD have been shown to be protective against radiation damage, and induction of MnSOD has been implicated in radiation-induced adaptive response [25], [26]. In recent years, MnSOD-based and Mn porphyrin-based therapeutic approaches have been designed/formulated to prevent radiation therapy-mediated damage to normal tissues [27], [28], [29], [30], [31], [32]. The effective protection has been demonstrated in esophagus, lung, oral cavity, small intestine, and also in whole body irradiation [27], . Because the bulk of ROS from normal cellular metabolic process is generated in the mitochondria, MnSOD plays an important role in the maintenance of normal mitochondrial functions and mitochondria structural integrity. Consequently, mutant mice with complete absence of MnSOD suffer from severe mitochondrial defects, predominantly in the brain and the heart [37], [38], [39].

MnSOD activities change with different stages of cell proliferation and differentiation [40], [41], [42], [43]. In general, MnSOD levels are lower in undifferentiated cells, and increase as cells differentiate. High levels of MnSOD have been shown to increase cell doubling time, while reduced levels are associated with a shorter cell cycle [40], [41], [42], [44]. Consistent with the important role of MnSOD in cell differentiation and proliferation, an earlier study showed that a 50% MnSOD deficiency negatively impacted the generation of new neurons in the dentate gyrus of the hippocampus [45]. On the other hand, MnSOD mutant mice were more resistant to irradiation-induced reduction in hippocampal neurogenesis, and as a consequence, the mutant mice had a higher level of hippocampal neurogenesis when compared to the irradiated wild type controls [45]. The mechanism for this paradoxical ″non-responsiveness″ to irradiation was not clear, but may involve a type of adaptive response leading to reduced radiosensitivity, enhanced progenitor cell proliferation, enhanced survival of newborn neurons, or the combination of the above. The neurocognitive consequence of this preserved hippocampal neurogenesis was also not clear. The work reported here addresses if altering the redox environment, by means of reduced MnSOD levels, prior to irradiation therapy will change the dynamics of hippocampal neurogenesis and the associated cognitive functions following irradiation.

## Materials and Methods

### Ethics Statement

All animal procedures were reviewed and approved by the Subcommittee on Animal Studies (NIH assurance number A3088-01) at the VA Palo Alto Health Care System and in accordance with the PHS Policy on Humane Care and Use of Laboratory Animals.

### Animals

Heterozygous *Sod2* knockout (*Sod2*−/+) and wild type (*Sod2*+/+) littermate controls were generated by crossing *Sod2*−/+ with C57BL/6J mice. All mice were housed in a barrier facility with a 12-hour dark-light cycle, given food and water *ad libitum*, and maintained in microisolators with a constant temperature between 20°C and 26°C.

### Cranial Irradiation

Two month old male *Sod2*+/+ and −/+ mice were anesthetized (IP injection, 120 mg/kg ketamine and 5 mg/kg xylazine) and sham irradiated or irradiated with a dose of 5 Gy gamma irradiation using a Mark 1 Cesium Irradiator (J.L. Shepherd and Associates, San Fernando, CA) with an exposure rate of 71.4 cGy/minute. The bodies of the mice were shielded with a minimum of 5 cm lead in all directions with the exception of the head, which was exposed to the irradiation through the opening of the lead shield.

### Bromodeoxyuridine Administration

**Figure 1:**
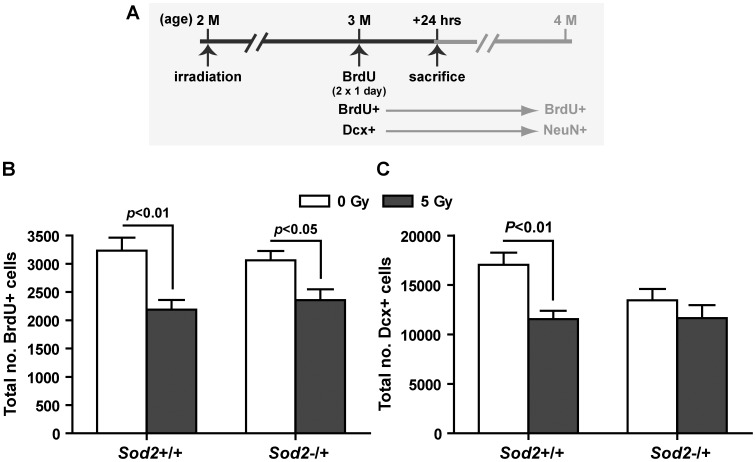
Number of proliferating cells and early lineage commitment in the SGZ. A, experimental timeline. Cells were labeled with BrdU at 4 weeks after irradiation (i.e. at 3 months of age) with two injections (8 hours apart) and sacrificed 24 hours after the first injection. The expected cell identities (BrdU+ → BrdU+; Dcx+ → NeuN+), if were allowed to continue the maturation process for another month, were also depicted. B, the number of proliferating cells (BrdU+) in the SGZ. C, the number of immature neurons (Dcx+) in the SGZ. Two-way ANOVA with Bonferroni post test was carried out. Radiation was the major source of variation in BrdU (*F*_(1,21)_ = 20.13, p = 0.0002) and Dcx (*F*_(1,16)_ = 9.30, p = 0.0077) analyses, and there was no genotype×treatment (radiation) interaction. P values indicate results from Bonferroni post tests. Data are presented as mean ± SEM. Sample size (in the order of *Sod2*+/+/0 Gy, *Sod2*+/+/5 Gy, *Sod2*−/+/0 Gy, and *Sod2*−/+/5 Gy): BrdU, n = 6, 5, 7, and 7; Dcx, n = 6, 5, 4, and 5.

**Figure 2:**
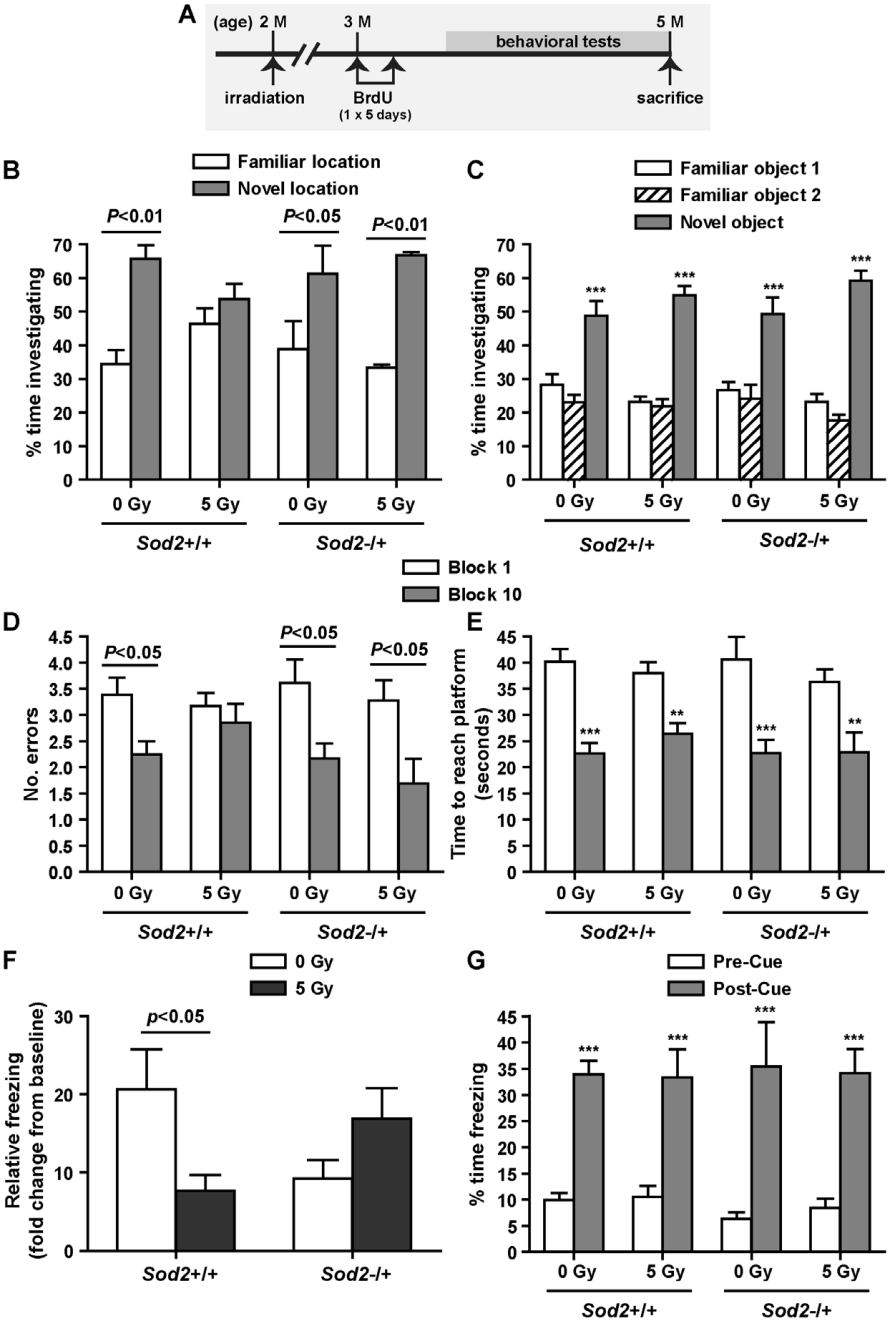
Hippocampal-dependent learning and memory. A, experimental timeline. Cells were labeled with BrdU at 4 weeks after irradiation (1 injection per day×5 days), evaluated for neurocognitive functions, and sacrificed after the completion of behavioral studies. B, novel location recognition task; comparison of time spent investigating an object in a familiar vs. a novel location. C, novel object recognition task; comparison of time spent investigating familiar objects vs. a novel object. D, radial-arm water maze; comparison of errors in arm entry in the beginning (block 1) vs. the end (block 10) of the test. E, radial-arm water maze; comparison of time spent finding the platform in the beginning (block 1) vs. the end (block 10) of the test. F, contextual fear condition; relative freezing to the baseline on day 1. G, cued conditioning; comparison of % time freezing in a novel environment during the pre-cue vs. the post-cue phase. P values from the Bonferroni post test are shown. ***, p<0.001. Data are presented as mean ± SEM. Sample size (in the order of *Sod2*+/+/0 Gy, *Sod2*+/+/5 Gy, *Sod2*−/+/0 Gy, and *Sod2*−/+/5 Gy): n = 5, 6, 5, and 4 for novel location recognition; n = 13, 12, 8, and 9 for novel object recognition; n = 18, 18, 13, and 14 for radial-arm water maze and contextual fear tests.

To label proliferating cells, the thymidine analog 5-bromo-2′-deoxyuridine (BrdU, Sigma, St Louis, MO) was prepared in PBS and administered (IP, 50 mg/kg) twice in one day with an eight-hour interval (short-term), or once per day for 5 contiguous days (long-term) beginning one month after irradiation. To determine the number of proliferating cells in the subgranular zone (SGZ) of hippocampal dentate gyrus one month after irradiation, the short-term protocol was implemented and animals were sacrificed 24 hrs after the first injection (Figure 1A). To assess long-term survival of newborn cells after behavioral studies, the long-term protocol was implemented one month after irradiation and animals were sacrificed 7 weeks after the final BrdU injection (Figure 2A).

### Cell Counting

The stereological counting principle of systematic, uniformly random sampling of sections was applied to determining the total number of BrdU positive (BrdU+) and doublecortin positive (Dcx+, cell marker for immature neurons) cells [46]. Mice were deeply anesthetized with ketamine and xylazine and perfused transcardially with 0.9% NaCl. Brains were removed and post fixed with 4% (w/v) PBS buffered paraformaldehyde for 48 hrs, and then immersed in 30% sucrose (in PBS) for cryoprotection. Forty-µm serial coronal sections encompassing the entire hippocampal formation were obtained with a sliding microtome (Leica SM 2000R, Leica, Mannheim, Germany). Free floating brain sections were stored in 1.5 mL Eppendorf tubes at −20°C in cryoprotective solution containing 0.01 M NaH_2_PO_4_, 30% glycerin, and 30% ethylene glycol.

For the detection of BrdU signals, brain sections were washed in Tris-buffered Tween solution (TBST) and then treated with 0.6% hydrogen peroxide and 0.1% Triton X-100 at room temperature (RT) for 30 min. To denature DNA, sections were treated with 3 M hydrochloric acid (HCl) at 37°C for 30 min. Sections were then incubated sequentially with a blocking solution containing 10% rabbit serum in TBST for 1 hr at RT, rat anti-BrdU (ab6326, 1∶1000 diluted in blocking solution, Abcam PLC, Cambridge, MA) over night, and biotinylated rabbit anti-rat IgG (BA-4000, 1∶1000, Vector Laboratories, Inc, Burlingame, CA). For the detection of doublecortin (goat anti-DCX, sc-8066, 1∶300, Santa Cruz Biotechnology, CA), brain sections were washed in TBST and pretreated for 30 minutes with 0.1% Triton and 0.6% H_2_O_2_ in TBST prior to incubating with primary antibody for 48 hrs at 4°C followed by biotinylated rabbit anti-goat IgG (BA-5000, 1∶1000, Vector Laboratories). After one hour incubation with the secondary antibody at RT, sections were treated with the ABC kit (PK-4000, Vector Laboratories) for 30 min at RT according to the manufacturer’s instruction. Diaminobenzidine (DAB, D5905, Sigma, St. Louis, MO) was used to identify BrdU+ or Dcx+ cells. Sections were mounted with Entellan (Electron Microscopy Sciences, Hatfield, PA) and stored at RT.

BrdU+ cells in the subgranular zone (SGZ) and the granular cell layer (GCL) of the dentate gyrus were quantified. Any positively stained cells appearing within 2 times the nuclear diameter away from the border of granule cell layer and hilus were considered as part of the SGZ, all other positively labeled cells in the dentate were considered as part of the GCL. To avoid over estimation, only BrdU signals that could be identified as constituting the middle cross-section of a nucleus were counted. Spot-like patterns from small patches of chromatin/chromosomes were not considered. Similar to BrdU analysis, Dcx+ cells were counted in the SGZ. To count BrdU+ and Dcx+ cells, the entire dentate gyrus (bilateral) of each brain section was photographed (Olympus Microscope, UPlan Apo, 10x/0.4) on an Olympus BX51 bright field light microscope connected to a SPOT FLEX™ camera (Diagnostic Instruments, Inc, Sterling Heights, MI). BrdU+ and Dcx+ cells were counted in one of every 6^th^ sections (8–10 slices total) of the hippocampal formation and the sum was multiplied by 6 to determine total number of BrdU+ and Dcx+ cells in the SGZ.

### Cell Fate Decision

To determine cell fate decisions in the SGZ, triple immunofluorescence staining of BrdU/NeuN/GFAP was carried out. Brain sections were rinsed in TBST, incubated in 3 M HCl for 30 min at 37°C to allow DNA denaturation, and rinsed again in TBST. Sections were incubated simultaneously with rat anti-BrdU (1∶1000), mouse anti-NeuN (MAB377, 1∶500, Chemicon, Temecula, CA), and rabbit anti-GFAP (Z0334, 1∶1000, DAKO, Carpinteria, CA) in TBST containing 10% goat serum overnight at 4°C followed by incubation with secondary antibodies (Alexa 555 goat anti-rat IgG, Alexa 488 goat anti-mouse IgG, and Alexa 647 goat anti-rabbit IgG, all at 1∶500 dilution, Invitrogen, Carlsbad, CA), at RT for 1 hr. Sections were mounted with ProLong® antifade (P36935, Invitrogen) solution and stored in −20°C. Immunofluorescence stained sections were analyzed with a LSM 510 Confocal Laser Scanning Microscope (Carl Zeiss MicroImaging, Zeiss EC Plan-Neofluar, 20x/0.05) with the detection pinhole set at 1 Airy Unit. Z sections taken at 3 µm intervals were examined for each BrdU+ cell with split panel analysis. For each lineage-specific marker, the percentage of BrdU+ cells expressing that marker was determined. For most samples, one of every 12^th^ sections was analyzed and at least 50 BrdU+ cells were examined for lineage analysis. Total number of lineage-specific BrdU+ cells for each animal was then calculated by multiplying the percentage by the total number of BrdU+ cells in the SGZ.

### Golgi Staining

Due to different requirements for tissue processing, a subset of experimental animals was used for Golgi staining. Ninety minutes following completion of the behavioral tasks animals were deeply anesthetized with ketamine and xylazine and then sacrificed by cervical dislocation. Brains were removed and bisected down the midline. Half of each brain was dissected and used for western blot analyses. The other half was processed using the Modified Golgi-Cox staining kit (Weill Cornell Medical College, New York, NY). Briefly, tissues were immersed in Golgi stain solution for 7 days with an initial change of solution after the first 12 hours. Brains were then transferred to a 30% sucrose solution and incubated for 3 days at 4°C with the solution changed after the first 12 hours. Tissues were then embedded in a 3% agarose solution and cooled prior to slicing on a Leica VT 1200S vibratome (Leica Microsystems, Bannockburg, IL). Samples were sectioned at 125 µm and mounted with 0.3% gelatin. Mounted sections were then brushed with a 50% sucrose solution and allowed to air dry overnight in a dark place. Slides were then washed in ddH_2_O 3 times for 5 minutes each with gentle shaking, transferred to the Blackening Solution for 10 minutes, then washed again with ddH_2_O 3 times for 5 minutes each. Slides were dehydrated through graded ethanol solutions, cleared with xylene, and coverslipped using Permount. Representative images of secondary and tertiary projections from dentate granular neurons were acquired using a 100x objective lens with a Zeiss Axioimager D1 microscope (Carl Zeiss MicroImaging, GmBH Germany) and Hamamatsu Orca-ER (C4742-80) digital camera (Hamamatsu Photonics, Bridgewater, NJ, USA). Dendritic spines were counted and lengths of neurites were measured using Image J software. Only spines with clear neck and head structures were counted.

### Measurements of Oxidative Stress

Lipid peroxidation and protein nitration were used as indices of oxidative stress in the hippocampus and were determined at 1 and 3 months following cranial irradiation in sham and irradiated *Sod2*+/+ and *Sod2*−/+ mice. The advanced lipid peroxidation end product, stable 4-hydroxynonenal (4-HNE) adducts, was used as a measurement of lipid peroxidation; 3-nitrotyrosine (3-NT) was used to determine the level of protein nitration. Total tissue lysates from hippocampus were prepared at 10 µg/ml and 20 µg/ml for 4-HNE adducts and 3-NT immunoassays, respectively. The commercial 4-HNE adducts and 3-nitrotyrosine ELISA kits were used (Cell Biolabs, Inc., San Diego, CA) for the assays, and results were compared to standard curves established with HNE-BSA and nitrated BSA, respectively.

### Western Blot Analyses

Protein levels of MnSOD, voltage-dependent anion channel (VDAC), and AP endonuclease 1 (APEX) in the hippocampus were determined by western blot analyses. VDAC is a mitochondria structural protein located in the outer membrane and the level of VDAC can be used to reflect the level of mitochondrial mass [47]. APEX is a dual function protein capable of repairing oxidative DNA damage (AP endonuclease function), and it also functions as a redox factor (Ref-1) to enhance the DNA binding capacity of a number of transcription factors [48]. Hippocampal tissues from each animal were homogenized individually (weight to volume ratio 1∶10) in T-PER buffer (Thermo Scientific, Rockford, IL) containing Complete® protease inhibitors and phosphatase inhibitors (Roche, Switzerland). Homogenized samples were centrifuged at 10,000x g at 4°C for 5 minutes, and the supernatants were stored in 20 µl aliquots at −80°C. Protein concentration of each sample was determined with a NanoVue spectrophotometer (GE Healthcare, Pittsburgh, PA).

Equal amounts of protein (70 µg) from each sample were separated by Mini-Protean TGX Any kD™ precast gel (Bio-Rad 456-9036) and transferred to nitrocellulose membranes. The following primary antibodies were used: MnSOD (rabbit polyclonal, 1∶3,000 dilution, Stressgen, SOD-110), VDAC (rabbit polyclonal, 1∶500 dilution, Abcam, ab15895), APEX (mouse monoclonal, 1∶1,000 dilution, Novus, NB100–116). Protein bands were then identified using appropriate secondary antibodies (goat anti-mouse IR680, goat anti-mouse IR800, or goat anti-rabbit IR680, 1∶20,000 dilution, Li-Cor Biosciences, Lincoln, Nebraska, USA) and visualized using an Odyssey® Infrared Imaging System (Li-Cor). All blots were re-probed with an antibody against β-actin (mouse monoclonal, 1∶5,000 dilution, Sigma, A5060) as a loading control. Quantification of western blot results was performed by normalizing the signal intensity of each sample to that of β-actin. A common brain sample was also loaded in all gels to allow signal normalization across multiple membranes.

### Behavioral Tests

Behavioral tests were started 10 days after the last BrdU injection (Figure 2A). The tests were carried out sequentially in the order described below and took five and half weeks to complete. Before the behavioral tests, each mouse was habituated to handling by lab personnel for 1 to 2 minutes each day for 3 or 4 days. Seventy% ethanol was used for cleaning and served as a mild olfactory cue in open field, elevated zero maze, and in the conditioning chamber of the contextual fear conditioning test; 4% acetic acid was used as the olfactory cue, following cleaning with water first, in the testing arena for novel object recognition, novel location recognition, and the cued conditioning chamber of the contextual fear conditioning test. Because 4% acetic acid had a strong scent, it also helped to mask residual odors left from previous trials. All behavioral studies were captured by a digital camera and the study results analyzed by TopScan Lite (CleverSys, Inc. Reston, VA), FreezeScan (CleverSys, Inc.), or by hand.

#### 1) Open field

The open field arenas were constructed with white opaque high-density polyethylene plastic and each measured 45×45×45 cm. The entire box was cleaned with 70% alcohol between each test subject. Each mouse was placed into the arena from the middle of the North edge under dim light and allowed to explore the box for 10 min. The time spent in the center 50% area (a virtual area defined by the software approximately 32×32 cm^2^ in the center of the open field) was used as an index of general anxiety levels. Total distance traveled and average velocities were measured to assess locomotor activities.

#### 2) Novel object recognition

The novel object recognition task was completed as previously described [49] with minor changes. Twenty-four hours following the open field task, each mouse was habituated for ten minutes in an open field arena that contained three identical objects. Objects were place 15 cm from 3 of the 4 corners. Twenty-four hours later, animals were returned to the arena and allowed to investigate replica objects in three 10-minute trials. For each trial, the time spent exploring the objects was recorded. After the third trial, one of the familiar objects was replaced with a novel object and animals were returned and allowed to investigate for another 10 minutes. There were 5-minute intervals between all trials, which allowed for cleaning (water, followed by 4% acetic acid) of the arenas and replacing objects with replicas to remove any olfactory cues. Animals were returned to their home cages during this interval. The total time spent exploring all objects was compared between trails to determine the familiarization of each mouse with the objects. The percent time spent exploring the novel object during the final trial was calculated and compared to the amount of time spent investigating the familiar objects in order to assess novel object recognition. The percent time spent exploring the objects were then compared based on genotype and treatment.

#### 3) Novel location recognition

The novel location recognition task was completed as previously described [49] with minor changes. This task was carried out in an open field arena with the addition of a large (21.5 cm×28 cm) patterned visual cue on the South wall. Twenty-four hours following the open field task, each mouse was habituated to the arena with the visual cue and two identical objects for 10 min. Objects were placed parallel to each other 30 cm from the cue. Twenty-four hours later animals were returned to the arena and allowed to investigate replica objects and the same visual cue for three 10-minute trials. For each trial, the time spent exploring the objects was recorded. Following the third trial, one of the familiar objects was moved 10 cm away from the visual cue to evaluate hippocampal-dependent novel location recognition. The inter-trial intervals were 5 minutes each. The total time spent exploring all objects was compared between trails to determine the familiarization of each mouse with the objects. Percent time investigating Object 1 (object in the novel location) was expressed as a percentage of total time spent investigating all objects in trial 4. The percent time spent exploring the objects were then compared based on genotype and treatment.

#### 4) Elevated zero maze

The zero maze was elevated 60 cm above the ground. The maze consisted of 4 equally sized quadrants with two opposing walled arms and two opposing open arms. The walls were 15 cm tall. The inner diameter of the circle was 50 cm and the outer diameter was 60 cm. This allowed for a 5-cm wide continuous circle. Mice were placed facing into closed quadrant 1 to begin the 5-min test period. The time spent in each of the four quadrants was measured and total time spent in open quadrants was used as indications of general anxiety. After mice were tested the chamber was cleaned with 70% ethanol and allowed to dry.

#### 5) Radial-arm water maze

The radial-arm water maze is a hippocampal-dependent task which has been described at length in previous publications [50], [51]. A slight modification was applied in the current radial-arm water maze task. Briefly, the radial-arm water maze consists of a metal round tank (diameter, 168 cm; height, 56 cm; depth, 43 cm) filled with water (22°C) mixed with non-toxic white paint. Six V-shaped stainless steel inserts (height, 54 cm; length, 56 cm) were placed in the tank to produce six swim arms radiating from an open central area. A large visual cue (30 x 46 cm) with contrasting colors was placed on each of the north, south, east and west walls of the experimental area. A red, plastic platform (12×8 cm) placed 1 cm above the surface of the water at the end of one arm (referred to as the “goal arm”) was used as the visible platform; a clear transparent plastic platform (12×8 cm) placed in the same goal arm at 0.5 cm below the surface of the water was used as the hidden platform. At the start of each trial, mice were released into one arm (referred to as the “start arm”) facing the center of the maze. The start arm was changed in each trial in a pre-determined pseudorandom fashion. If a mouse did not locate the platform within 1 min, it was gently guided to the platform by the experimenter. Once a mouse found or was guided to the platform, it was left undisturbed on the platform for 15 s. Mice received a total of 15 trials on each of the training and test day, including two 30-min breaks following trials 6 and 12. During the break period, the mice were returned to their home cages. On the first day (training day), trial 1 to 12 alternated between visible and hidden platforms, while trial 13 to 15 used hidden platform only. On the second day (test day), mice were given 15 trials of the hidden platform test in the radial-arm water maze to assess their memory for the hidden platform location. Spatial learning and memory was measured by counting both the number of arm entry errors and time spent to find the platform for each trial. An arm entry error was objectively defined as an entry into one of the arms that did not contain the platform. Average arm entry errors and time spent finding the platform were calculated for blocks of three trials.

#### 6) Fear conditioning

Fear conditioning was used to evaluate both hippocampal-dependent and hippocampus-independent learning and memory. Briefly, mice were placed into the conditioning chamber and allowed to explore for five minutes in order to habituate the animal to the environment and collect baseline freezing levels using FreezeScan (CleverSys, Inc). Operant conditioning chambers (26×32×21 cm) were housed in sound attenuating chambers (43.2×45.7×43.2 cm). All chambers were cleaned using a 70% ethanol solution before each session. After habituation, mice were then presented with a tone and cue light for 30 seconds (conditioned stimulus - CS). A mild foot shock (0.4 mA) was paired for the last 2 seconds of the tone/cue light presentation (unconditioned stimulus - US). The mice were then allowed to investigate the environment for an additional three minutes before being exposed to the CS-US pairing a second time. Thirty seconds after the second CS-US pairing, animals were removed to their home cages. Twenty four hours following their training session, animals were returned to the training chambers and allowed to investigate for 5 minutes. The hippocampal-dependent contextual fear response was scored as a ratio of time spent freezing on day 2 relative to baseline freezing on day one in one minute bins. To test hippocampus-independent memory function, opaque, white floor and wall inserts were added to change the appearance of the conditioning chamber. Inserts were wiped with a 4% acetic acid solution to clean and change the olfactory cue. One hour following re-exposure to the conditioning chambers, animals were placed in the modified chambers and freezing behavior was scored for 3 minutes. Then the tone and cue lights were presented for 30 seconds without a pairing of the foot shock. Freezing behavior was measured during the three minutes following the CS in the new environment.

### Statistical Analyses

All statistical analyses were completed using GraphPad Prism 4 (La Jolla, CA, USA). For the genotype (*Sod2*+/+ vs. *Sod2*−/+)×treatment (sham vs. irradiation) or treatment×time (before vs. post behavioral tests) analyses, two-way ANOVA with Bonferroni post test was carried out. For comparison among different experimental groups, one-way ANOVA with Tukey’s post test was performed.

## Results

### Progenitor Cell Proliferation and Differentiation Before Behavioral Studies

To assess the extent of progenitor cell proliferation, the number of BrdU+ cells in the SGZ was determined following a short-term BrdU labeling protocol (Figure 1A) carried out at one month after irradiation. No significant difference in the number of BrdU+ cells in the SGZ was observed between sham irradiated *Sod2*+/+ and *Sod2*−/+ mice (mean ± SEM, 3234±231 vs. 3061±168), while significant suppression of progenitor cell proliferation was observed in both irradiated *Sod2+/+* (32% reduction, t = 3.58, p<0.01) and *Sod2−/+* mice (23% reduction, t = 2.72, p<0.05) (Figure 1B). Additionally, the number of BrdU+ cells in the SGZ was not significantly different between the two irradiated groups (2188±173 vs. 2358±190).

In contrast, the number of immature neurons (Dcx+ cells) in sham irradiated *Sod2*−/+ mice was 21% lower than that in sham irradiated *Sod2*+/+ mice (*Sod2*+/+ vs. *Sod2*−/+; 17036±1244 vs. 13436±1159), although the difference did not reach statistical significance (Bonferroni post test, t = 2.11, p>0.05) (Figure 1C). Similar to the BrdU data, a 32% reduction (t = 3.43, p<0.01) in the number of immature neurons was observed in the irradiated *Sod2*+/+ mice (Figure 1C). However, no significant reduction in the number of Dcx+ cells was observed in irradiated *Sod2*−/+ mice. Despite differences in the MnSOD level, the number of Dcx+ cells was not significantly different between irradiated *Sod2*+/+ and *Sod2*−/+ mice (11545±852 vs. 11636±1322).

### Anxiety and Motor Activities

Since anxiety can be a strong confounding factor in the outcome of learning and memory tests, open field and elevated zero maze studies were performed. Neither genotype nor irradiation had significant effects on anxiety-like behaviors seen in an open field test or elevated zero maze (data not shown). When given the opportunity to explore the elevated zero maze, animals spent 22–26% of the allotted 5 minutes in the open quadrants with no significant differences between genotype or treatment. In addition, all animals spent 13–14% of a 10-minute time period exploring in the center fifty percent area of the low-light open field arena (data not shown). Total distance traveled and average velocity also showed no significant differences between groups, indicating no impairment in locomotor activities (data not shown).

### Hippocampal-dependent Learning and Memory Behaviors

#### 1) Novel location and novel object recognition

Irradiation significantly impaired the abilities of *Sod2*+/+ mice to determine when a familiar object had been moved to a novel location (Figure 2B). Sham irradiated *Sod2*+/+ mice were able to determine novel placement of a familiar object (t = 4.03, p<0.01), whereas their irradiated counterparts lost this ability (t = 1.02, p>0.05). However, both sham irradiated (t = 2.89, p<0.05) and irradiated *Sod2*−/+ mice (t = 3.85, p<0.01) significantly increased investigation of the object in the novel location, suggesting that irradiation had no effects on *Sod2*−/+ mice in spatial recognition. All groups were capable of discerning when a novel object replaced a familiar object (*F*_(2,111)_ = 119.9, p<0.0001) (Figure 2C).

#### 2) Radial-arm water maze

The number of errors in finding the platform were not significantly reduced in irradiated *Sod2*+/+ mice after 30 trials in the radial-arm water maze task (t = 0.51, p>0.05) (Figure 2D). In comparison, the number of errors (t = 2.87, p<0.05) and time (t = 5.40, p<0.001) in finding the platform was significantly reduced in sham irradiated *Sod2*+/+ mice. Similar results were observed in both sham irradiated and irradiated *Sod2*−/+ mice in the number of errors (t = 2.70, p<0.05; t = 2.96, p<0.05) and time (t = 5.03, p<0.001; t = 4.81, p<0.001) in the radial-arm water maze task (Figure 2D and 2E).

#### 3) Fear conditioning

When re-exposed to only the training environment for fear conditioning without presentation of the conditioned stimuli (tone and light cues), irradiated *Sod2*+/+ mice showed less relative freezing in the first minute (t = 2.65, p<0.05) compared to the sham irradiated *Sod2*+/+ mice (Figure 2F). In contrast, irradiated *Sod2*−/+ mice maintained freezing at a similar level to their sham irradiated counterparts (t = 1.34, p>0.05) upon re-exposure to the training context. However, when the training environment was altered structurally in combination with an alternative olfactory cue (4% acetic acid), all animals showed significant freezing responses to the conditioned stimuli (*F*_(1,110)_ = 82.38, p<0.0001) (Figure 2G).

### Long-term Survival of New Neurons after Behavioral Studies

**Figure 3:**
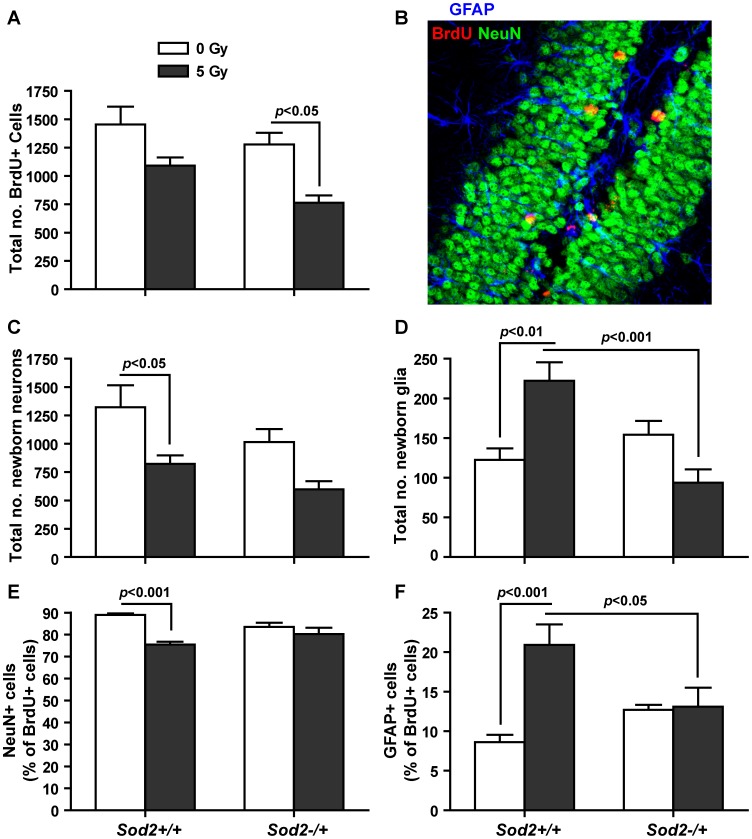
Long-term survival of newly generated cells in the SGZ following behavioral studies. A, total number of BrdU+ cells in the SGZ. *Sod2* genotype (*F*_(1,23)_ = 4.71, p = 0.041) and radiation treatment (*F*_(1,23)_ = 14.28, p = 0.001) both contributed significantly to variations in BrdU numbers, but there was no interaction between genotype and treatment (*F*_(1,23)_ = 0.44, p = 0.52). B, a representative image showing BrdU+/NeuN+ and BrdU+/GFAP+ cells in the SGZ of the dentate gyrus. C, the total number of newborn neurons. Genotype (*F*_(1,16)_ = 4.66, p = 0.046) and treatment (*F*_(1,16)_ = 13.76, p = 0.0019) both contributed significantly to variations in BrdU+/NeuN+ numbers, but there was no interaction between these two factors. D, the total number of newborn glial cells. Only genotype (*F*_(1,16)_ = 6.97, p = 0.018) contributed significantly in the data variation, and there was an interaction between genotype and treatment (*F*_(1,16)_ = 19.05, p = 0.0005). E, the percentage of newly born neurons. Only treatment (*F*_(1,16)_ = 19.80, p = 0.0004) contributed significantly to the data variation, and there was an interaction between genotype and treatment (*F*_(1,16)_ = 7.30, p = 0.0157). F, the percentage of newly born glial cells. Only treatment (*F*_(1,16)_ = 11.79, p = 0.0034) contributed significantly to the data variations, and there was an interaction between genotype and treatment (*F*_(1,16)_ = 10.27, p = 0.0055). Data are presented as mean ± SEM. Sample size (in the order of *Sod2*+/+/0 Gy, *Sod2*+/+/5 Gy, *Sod2*−/+/0 Gy, and *Sod2*−/+/5 Gy): BrdU, n = 8, 6, 7, and 6; BrdU+/NeuN+ and BrdU+/GFAP+, n = 5 each.

To determine the level of neurogenesis following behavioral studies in the post irradiation environment, a long-term BrdU labeling protocol was carried out prior to the behavior tests, and the number of BrdU+, BrdU+/NeuN+, and BrdU+/GFAP+ cells in the SGZ were determined after the completion of behavioral studies (Figure 2A). Even though the BrdU labeling procedures were different (short-term vs. long-term protocol), the BrdU pattern, in terms of effects of *Sod2* genotype and irradiation, measured after the completion of behavioral studies was similar to that measured before behavioral studies (Figure 1B) and showed no significant difference in the number of BrdU+ cells between sham irradiated *Sod2*+/+ and *Sod2*−/+ mice (1454±156 vs. 1279±101) (Figure 3A). Compared to sham irradiated controls, irradiated *Sod2+/+* animals showed a 25% reduction in the number of BrdU+ cells following behavior studies; however, the difference was not statistically significant (t = 2.24, p>0.05). On the other hand, the number of BrdU+ cells in the SGZ of irradiated *Sod2*−/+ mice was significantly reduced (40% reduction, t = 3.09, p<0.05) from that of sham irradiated *Sod2*−/+ following behavioral studies (Figure 3A).

When the proportion of newborn neurons (BrdU+/NeuN+) and astroglia (BrdU+/GFAP+) were determined by double labeling (Figure 3B), the number of new neurons in the SGZ of sham irradiated *Sod2*−/+ mice was 77% that of sham irradiated *Sod2*+/+ (t = 1.76, p>0.05) (Figure 3C). Following behavioral studies, the number of BrdU+/NeuN+ cells in irradiated *Sod2*+/+ mice was 38% lower (t = 2.85, p<0.05) than that in sham irradiated *Sod2*+/+ controls. These results mirrored the level of Dcx+ cells determined before behavioral studies (Figure 1C), and suggested the same general response to MnSOD deficiency and irradiation in sham irradiated *Sod2*−/+ and irradiated *Sod2*+/+, respectively. On the other hand, whereas there was only a 13% difference in the number of Dcx+ cells between sham irradiated and irradiated *Sod2*−/+ mice before behavioral studies, there was a 41% reduction in the number of newly born neurons in irradiated *Sod2*−/+ mice after behavioral studies (t = 2.39, p>0.05; unpaired t test, p = 0.0148). The data suggested a possible interaction between irradiation and behavioral training in the MnSOD deficient environment.

Although fewer BrdU+ cells in total were seen in irradiated *Sod2−/+* mice following behavioral studies, the relative cell fate distributions between neurons and astrocytes were not significantly changed (Figure 3E and 3F). The irradiated *Sod2+/+* mice on the other hand, showed a significant decrease in the proportion of neurons generated (from 89% in sham irradiated to 75.5% of BrdU+ cells in irradiated *Sod2*+/+, t = 5.06, p<0.001) (Figure 3E) and an increase in the proportion of astrocytes generated (2.4-fold increase, t = 4.69, p<0.001) (Figure 3F). Two-way ANOVA analyses showed a significant interaction between genotype and treatment in the neuronal (*F*_(1,16)_ = 7.30, p = 0.0157) and glial (*F*_(1,16)_ = 10.27, p = 0.0055) lineage commitment.

### Dendritic Spine Density

**Figure 4:**
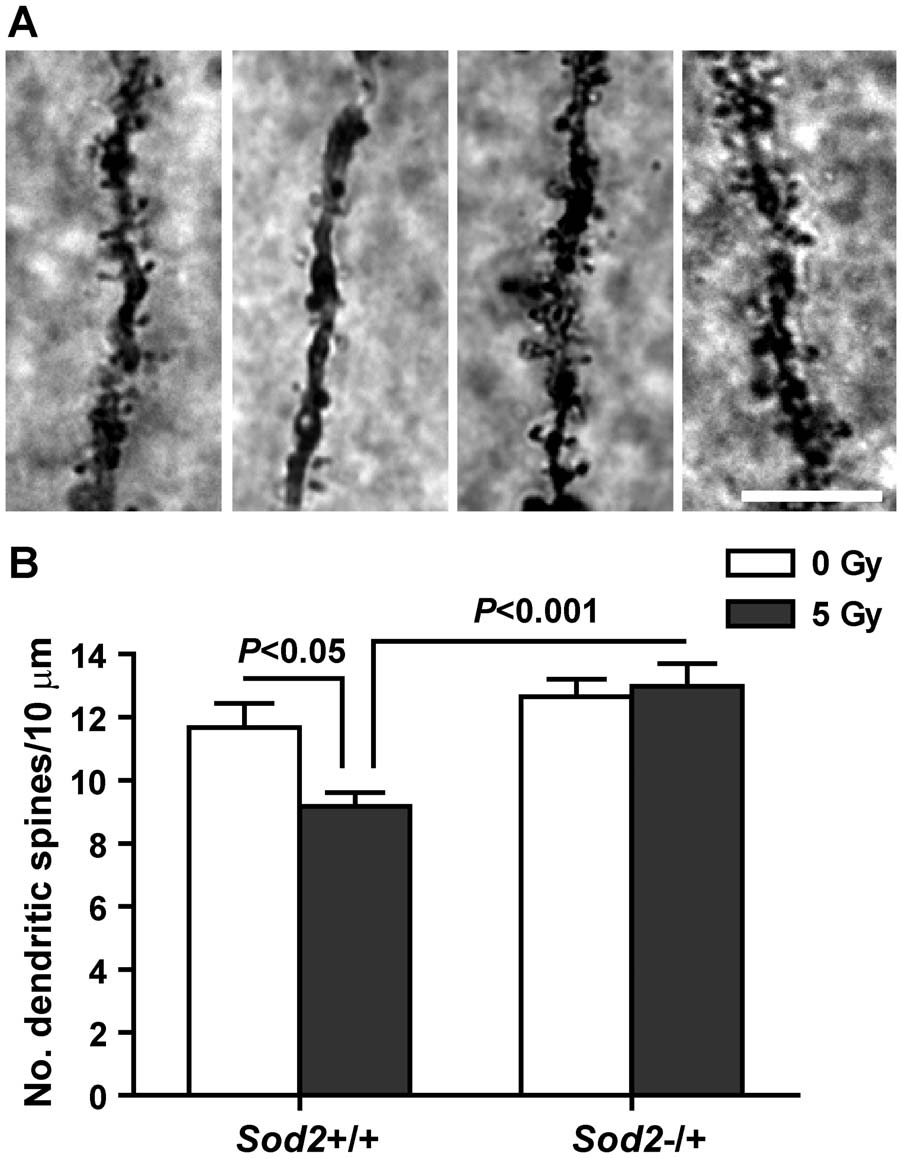
Dendritic spine density following behavioral studies. The number of dendritic spines was analyzed in secondary and tertiary dendrites of granule cells in the dentate gyrus after behavioral studies. Upper panel, representative photos from (left to right) *Sod2*+/+/0 Gy, *Sod2*+/+/5 Gy, *Sod2*−/+/0 Gy, and *Sod2*−/+/5 Gy mice. Lower panel, average spine densities. Sample size: n = 4, 6, 5, and 4 for *Sod2*+/+/0 Gy, *Sod2*+/+/5 Gy, *Sod2*−/+/0 Gy, and *Sod2*−/+/5 Gy, respectively. Only genotype (*F*_(1,15)_ = 15.59, p = 0.0013) contributed significantly to the data variations, and there was a significant interaction between genotype and treatment (*F*_(1,15)_ = 5.46, p = 0.0337). P values indicate Bonferroni post test results. Data are presented as mean ± SEM. Scale bar = 10 µm.

Dendritic spine densities were not significantly different between sham irradiated *Sod2*+/+ and *Sod2*−/+ mice after behavioral studies. However, spine densities were significantly lowered in irradiated *Sod2*+/+ mice analyzed after behavioral studies (21% reduction, t = 2.98, p<0.05) (Figure 4A and 4B). In contrast, irradiated *Sod2*−/+ mice showed no decrease in dendritic spine densities (t = 0.38, p>0.05) following behavioral studies when compared to sham irradiated *Sod2*−/+ controls (Figure 4B). Two-way ANOVA analysis showed a significant genotype×treatment interaction (*F*_(1,15)_ = 5.46, p = 0.0337) in the dendritic spine density.

### Oxidative Stress

**Figure 5:**
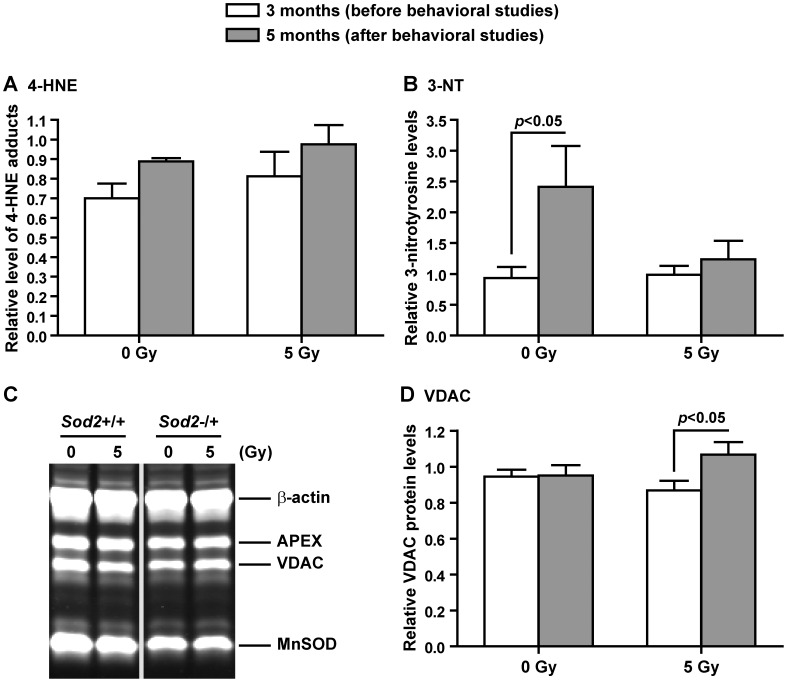
Redox state and mitochondrial mass. Levels of the advanced lipid peroxidation end product, 4-hydroxynonenal (4-HNE) adducts, (A) and protein nitration product 3-nitrotyrosine (3-NT) (B) were determined by ELISA to monitor the extent of oxidative stress in the hippocampus at 3 months and 5 months of age after a single dose of cranial irradiation (see timeline in Figure 3A). C, a representative western blot image from samples collected following behavioral studies showing APEX, VDAC, and MnSOD. D, relative protein levels of VDAC in the *Sod2*−/+ cohorts. In order to compare changes in 4-HNE, 3-NT, and VDAC levels in sham and irradiated *Sod2*−/+ from two different experimental stages – at 3 months of age before behavioral studies and at 5 months of age after behavioral studies – levels in *Sod2*−/+ mice were calculated as a percentage of their corresponding *Sod2*+/+ controls. Two-way ANOVA with Bonferroni post test was carried out. P values indicate Bonferroni post test results. There was no treatment×time interaction. Data are presented as mean ± SEM. Sample size: n = 5 each at 3 months of age, and 4 each at 5 months of age for sham and irradiated *Sod2*−/+.

To determine if radiation-induced oxidative stress persisted at the time when the neurogenesis and behavioral studies were performed, levels of lipid peroxidation (4-HNE adducts) and protein nitration (3-NT) in hippocampus were determined in tissues collected before (i.e. at 3 months of age) and immediately after (i.e. at 5 months of age) behavioral studies. No significant differences in the levels of 4-HNE adducts and 3-NT were observed between sham and the corresponding irradiated cohorts within each genotype and experimental group. There were also no significant changes in the levels of 4-HNE adducts following behavioral training (Figure 5A). In contrast, a significant increase in 3-NT levels in sham irradiated *Sod2*−/+ was observed following behavioral studies (t = 3.03, p<0.05) (Figure 5B); however, the 3-NT levels stayed relatively stable in irradiated *Sod2*−/+ following behavioral studies (Figure 5B).

### Western Blot Analyses

No induction of MnSOD by irradiation or behavioral training was observed in both sham and irradiated *Sod2*−/+ mice and the protein levels remained at 63–67% that of their corresponding *Sod2*+/+ controls (Figure 5C; quantification data not shown). MnSOD deficiency and irradiation also did not lead to significant changes of another mitochondrial protein, VDAC (Figure 5D). However, when looking at the relative changes in VDAC protein levels in samples collected before and after behavioral studies, the dynamics were different between sham and irradiated *Sod2*−/+ mice (Figure 5D). Whereas the protein levels in sham irradiated *Sod2*−/+ remained at 94–95% that of sham irradiated *Sod2*+/+, average VDAC protein levels in irradiated *Sod2*−/+ went from 87% to 107% of that in irradiated *Sod2*+/+ following behavioral studies (t = 2.57, p<0.05) (Figure 5D). Protein levels of APEX were not affected by *Sod2* genotype or irradiation, and the levels in sham and irradiated *Sod2*−/+ were measured at 94–100% of their corresponding *Sod2*+/+ controls before and following behavioral studies (data not shown).

## Discussion

In this report we showed that a 50% reduction in MnSOD altered baseline hippocampal neurogenesis and the response of these newly born cells to cranial irradiation in *Sod2*−/+ mice. Even though irradiated *Sod2*−/+ mice had a marked reduction in the number of new neurons following behavioral studies, these mice were able to maintain a similar level of performance as sham irradiated *Sod2*−/+ mice in studies designed to test the hippocampal-dependent functions of learning and memory. The ability to maintain normal dendritic spine densities in the dentate gyrus granule cells may be the key to unimpeded cognitive functions in *Sod2*−/+ mice following cranial irradiation.

Previous studies of hippocampal neurogenesis looking at long-term survival of newborn cells in the SGZ without the influence of behavioral tests showed a comparable number of BrdU+ cells and a roughly 33% reduction in newborn neurons in sham irradiated *Sod2*−/+ mice when compared to sham irradiated *Sod2*+/+ controls [45]. The study also showed a marked reduction in the number of BrdU+ cells and newly born neurons in irradiated *Sod2*+/+, but the numbers did not change significantly in irradiated *Sod2*−/+ mice [45]. In this follow-up study, we looked upstream in the experimental timeline (Figure 1) to determine if differences in progenitor cell proliferation and differentiation, or differences in the long-term survival of newly generated cells, dictated differences in the number of new neurons between *Sod2*+/+ and *Sod2*−/+ and between sham and irradiated groups observed at 4 months of age. Comparison of these two studies, taken with the caveats that these data were generated from two separate experiments and the counting criteria may not be identical, the results showed a consistent pattern in the effects of MnSOD deficiency, as well as the interaction between MnSOD deficiency and irradiation, on differentiation and long-term survival of newborn neurons.

First, MnSOD deficiency created a less favorable environment for neuronal differentiation (Figure 1C) without affecting progenitor cell proliferation (Figure 1B). Consequently, reduced numbers of immature neurons (Figure 1C) were observed in sham irradiated *Sod2*−/+ mice even though the level of progenitor cell proliferation (Figure 1B) was comparable to that of sham irradiated *Sod2*+/+ controls. In the next 4 weeks, a subpopulation of cells identified at that stage was expected to go through the maturation process, with the majority of them showing characteristics of mature neurons, i.e. BrdU+/NeuN+ (Figure 1A). Consistent with the differentiation timeline, the relative number of new neurons (BrdU+/NeuN+) identified at four months of age [45] mirrored that of Dcx+ cells identified at three months of age in sham irradiated *Sod2*+/+ and *Sod2*−/+ mice (Figure 1C). This comparison also suggested that MnSOD deficiency probably did not affect long-term survival of newly generated neurons. Second, MnSOD deficiency modified the response to irradiation and created a post irradiation neurogenic environment that was at least as favorable as its sham irradiated counterpart for the generation (Figure 1C) and long-term survival of new neurons [45]. The irradiated *Sod2*+/+ environment, in comparison, was less favorable for long-term survival of new neurons, and consequently, there was a disproportional decrease in BrdU+/NeuN+ cells at four months of age [45].

We previously showed that, similar to *Sod2*−/+ mice, irradiated mutant mice deficient in the extracellular superoxide dismutase (EC-SOD KO) were able to maintain the same level of neurogenesis as sham irradiated controls [16]. In addition, irradiated EC-SOD KO mice also performed well in behavioral studies designed to test hippocampal-dependent functions of learning and memory [49]. However, it was not clear if preserved neurogenesis in irradiated *Sod2*−/+ mice ([45] and Figure 1C) reflected unimpeded cognitive functions following irradiation. Three hippocampal-dependent spatial learning tests, novel location recognition, radial-arm water maze, and contextual fear conditioning, were performed and the results consistently showed defects in spatial recognition in irradiated *Sod2*+/+ mice, but not in irradiated *Sod2*−/+. The data suggest that similar to EC-SOD KO mice, preserved neurogenesis in *Sod2*−/+ mice after irradiation also correlates positively to intact cognitive functions. Similar to the previous study, non-hippocampal-dependent functions were not affected by irradiation and all experimental groups performed equally well in novel object recognition and cued conditioning tests (Figure 2C and 2G).

Physical exercise and novel environments have been shown to stimulate or suppress neurogenesis, depending on whether animals regard the treatment as a form of stimulation or a source of anxiety [52], [53], [54], [55]. In this study, hippocampal neurogenesis was assessed after the completion of behavioral studies. Compared to the relative levels of BrdU+ and Dcx+ cells between *Sod2*+/+ and −/+ mice at 3 months of age (Figure 1B and 1C), as well as data generated from the previous study without behavioral tests [45], there appeared to be a disproportional reduction in BrdU+ and BrdU+/NeuN+ cells in irradiated *Sod2*−/+ mice after the behavioral studies (41% reduction, Figure 3A and 3C). It was possible that, although irradiated *Sod2*−/+ mice performed well in the hippocampal-dependent tasks, the protracted behavioral training and testing imposed more stress on this group of mice. Previous studies suggested that irradiated or more oxidized environment reduced progenitor cell differentiation towards the neuronal lineage and instead, the environment favored the glial lineage [11], [16], [18]. Although not all comparisons reached statistically significant levels, irradiation and MnSOD deficiency drove the population of newborn neurons from 89% of total BrdU+ cells in sham irradiated *Sod2*+/+ mice to 75.5% and 83.5% in irradiated *Sod2*+/+ and sham irradiated *Sod2*−/+, respectively (Figure 3E). At the same time, the population of new astroglia changed from 8.6% of total BrdU+ cells in sham irradiated *Sod2*+/+ to 20.9% of that in irradiated *Sod2*+/+ mice (Figure 3F). In contrast, irradiated *Sod2*−/+ mice did not show a significant change from sham irradiated *Sod2*−/+ in the percentage of BrdU+/NeuN+ and BrdU+/GFAP+ populations (Figures 3E and 3F). These data suggest that the neurogenic environment in irradiated *Sod2*−/+ mice may have been altered to counter the negative effects of radiation on neuronal cell differentiation.

In this study, irradiated *Sod2*+/+ mice had a significant reduction in neurogenesis, and in parallel, showed deficits in spatial learning (Figure 2). In contrast, irradiated *Sod2*−/+ mice performed equally well as sham irradiated *Sod2*+/+ mice in all three learning and memory tests, even though the level of BrdU+/NeuN+ cells in irradiated *Sod2*−/+ was significantly lower than that in sham irradiated *Sod2*+/+ mice (one-way ANOVA with Tukey’s post test, p<0.01) at the end of behavioral studies (Figure 3C). Suppression of hippocampal neurogenesis can lead to deficits in hippocampal-dependent functions of learning and memory, whereas increased neurogenesis can lead to enhanced cognitive functions [12], [51], [56], [57]. However, there is not a strict correlation [58], [59], [60] because memory encoding is not limited to new neurons [9] and other factors, such as synaptic maintenance and synaptic plasticity, are at least equally, if not more, important in learning and memory [61]. Dendritic spines constitute the postsynaptic element of most excitatory synapses and mediate the majority of excitatory connections in the brain. Stable synaptic maintenance and connectivity are important for learning and memory [62], [63]. Loss of dendritic spines is an early event in mouse models of Alzheimer disease, and it correlates with synaptic loss and deficits in learning and memory [64], [65]. Irradiation and oxidative stress have also been shown to cause reduced dendritic spine densities in vitro and in vivo [66], [67], [68], [69], [70]. Consistent with the function of dendritic spines, irradiated *Sod2*+/+ mice showed defects in spatial learning and a significant reduction in dendritic spine densities in the dentate granule cells. At the same time, irradiated *Sod2*−/+ mice showed normal spatial learning and in parallel, there was no reduction in dendritic spine densities in this group of mice. Taken together, the data suggest that cognitive deficits in irradiated *Sod2*+/+ mice may have been caused by reduction in both neurogenesis and dendritic spine densities. On the other hand, in spite of lower number of new neurons at the end of behavioral studies, irradiated *Sod2*−/+ mice showed normal spatial learning, and this was probably due to a functional compensation from normal dendritic spine densities. The data also suggested that, under normal conditions, MnSOD deficiency did not lead to a reduction in dendritic spine density in dentate granule cells at least up to 5 months of age.

How MnSOD deficient mice managed to maintain normal dendritic spine density as well as normal cognitive function following irradiation is not fully understood at this time. Dendritic spine formation is normally controlled by multiple pathways [71], [72], [73], [74], [75], [76], and a recent study implicates an important connection between mitochondria and the TIAM1-Rac1GTP signaling pathway, which appears to be sensitive to oxygen free radicals [77]. Given the relationship between irradiation, MnSOD, and oxidative stress, altered redox state in the hippocampal environment may be an important confounding factor. Examination of the stable end products of lipid peroxidation and protein nitration, as a function of the hippocampal redox environment, showed no significant differences between sham and the corresponding irradiated cohorts (Figures 5A and 5B). However, the level of protein nitration increased significantly in sham irradiated *Sod2*−/+ mice but stayed relatively stable in irradiated *Sod2*−/+ following behavioral studies (Figure 5B). Higher levels of protein nitration suggest that there may be more production of peroxynitrite (ONOO^−^), which is the end product of NO and superoxide (O_2_^−^) [78]. Given that increased NO production is tightly linked to spatial learning [79], [80], higher levels of protein nitration in sham irradiated *Sod2*−/+ mice following behavioral studies implies that there may be more ONOO^−^ production under a MnSOD deficient environment. How irradiated *Sod2*−/+ mice managed to prevent increase in protein nitration was not clear, but it was probably not because of an up-regulation of antioxidant enzymes. An earlier study showed no changes in major antioxidant enzymes in irradiated *Sod2*−/+ mice at the time hippocampal neurogenesis was examined [45]. Consistent with that finding, no significant changes in MnSOD and APEX protein levels was observed in this study (Figure 5C).

In addition to the redox environment, mitochondrial oxidative phosphorylation is important for normal neuronal functions, including axonal transport and neurotransmitter release [81]. A positive correlation between the number of dendritic mitochondria and the number of synapses has also been reported [75]. The dynamic change in VDAC protein levels in irradiated *Sod2*−/+ mice (Figure 5D) suggested that there might be a parallel change in mitochondrial mass since VDAC was an integral part of mitochondrial structure. It was possible that the ability to dynamically adjust mitochondrial structural proteins in response to outside stimuli supported the normal maintenance of dendritic spine densities in irradiated *Sod2*−/+ mice. Consequently, irradiated *Sod2*−/+ mice were able to perform normally in behavioral studies designed to test hippocampal-dependent functions of learning and memory.

In summary, our studies showed that there may be a dynamic change in mitochondrial biogenesis in response to MnSOD deficiency, irradiation, and behavioral training, which collectively, led to a well-maintained dendritic system and unimpeded cognitive functions in spite of reduced generation of new neurons in the hippocampus of irradiated *Sod2*−/+ mice. The mechanism leading to dynamic changes of mitochondrial biogenesis in this experimental system is not clear and will require additional study in the future. Given the importance of mitochondria in neuronal functions and synaptic plasticities and the importance of MnSOD in the maintenance of mitochondrial functions and structural integrity, understanding how alterations in MnSOD affect hippocampal neurogenesis and dendritic maintenance, especially in the post-irradiation period, should provide a handle on how to manage the late effects of cranial irradiation and minimize the associated cognitive deficits.

## References

[1] Abayomi OK (1996) Pathogenesis of irradiation-induced cognitive dysfunction. Acta Oncol 35: 659–663. 8938210 10.3109/02841869609083995

[2] Laack NN, Brown PD (2004) Cognitive sequelae of brain radiation in adults. Semin Oncol 31: 702–713. 15497124 10.1053/j.seminoncol.2004.07.013

[3] Sarkissian V (2005) The sequelae of cranial irradiation on human cognition. Neurosci Lett 382: 118–123. 15911133 10.1016/j.neulet.2005.02.068

[4] Gondi V, Tome WA, Mehta MP (2010) Why avoid the hippocampus? A comprehensive review. Radiother Oncol 97: 370–376. 20970214 10.1016/j.radonc.2010.09.013 PMC2997490

[5] Eriksson PS, Perfilieva E, Bjork-Eriksson T, Alborn AM, Nordborg C, et al (1998) Neurogenesis in the adult human hippocampus. Nat Med 4: 1313–1317. 9809557 10.1038/3305

[6] Gage FH, Kempermann G, Palmer TD, Peterson DA, Ray J (1998) Multipotent progenitor cells in the adult dentate gyrus. J Neurobiol 36: 249–266. 9712308 10.1002/(sici)1097-4695(199808)36:2<249::aid-neu11>3.0.co;2-9

[7] Kempermann G, Gage FH (2000) Neurogenesis in the adult hippocampus. Novartis Found Symp 231: 220–235; discussion 235–241, 302–226. 11131541

[8] Aimone JB, Wiles J, Gage FH (2006) Potential role for adult neurogenesis in the encoding of time in new memories. Nat Neurosci 9: 723–727. 16732202 10.1038/nn1707

[9] Ramirez-Amaya V, Marrone DF, Gage FH, Worley PF, Barnes CA (2006) Integration of new neurons into functional neural networks. J Neurosci 26: 12237–12241. 17122048 10.1523/JNEUROSCI.2195-06.2006 PMC6675440

[10] Deng W, Aimone JB, Gage FH (2010) New neurons and new memories: how does adult hippocampal neurogenesis affect learning and memory? Nat Rev Neurosci 11: 339–350. 20354534 10.1038/nrn2822 PMC2886712

[11] Mizumatsu S, Monje ML, Morhardt DR, Rola R, Palmer TD, et al (2003) Extreme sensitivity of adult neurogenesis to low doses of X-irradiation. Cancer Res 63: 4021–4027. 12874001

[12] Christie LA, Acharya MM, Parihar VK, Nguyen A, Martirosian V, et al (2012) Impaired Cognitive Function and Hippocampal Neurogenesis following Cancer Chemotherapy. Clin Cancer Res 18: 1954–1965. 22338017 10.1158/1078-0432.CCR-11-2000

[13] Riley PA (1994) Free radicals in biology: oxidative stress and the effects of ionizing radiation. Int J Radiat Biol 65: 27–33. 7905906 10.1080/09553009414550041

[14] Limoli CL, Hartmann A, Shephard L, Yang CR, Boothman DA, et al (1998) Apoptosis, reproductive failure, and oxidative stress in Chinese hamster ovary cells with compromised genomic integrity. Cancer Res 58: 3712–3718. 9721883

[15] Panagiotakos G, Alshamy G, Chan B, Abrams R, Greenberg E, et al (2007) Long-term impact of radiation on the stem cell and oligodendrocyte precursors in the brain. PLoS One 2: e588. 17622341 10.1371/journal.pone.0000588 PMC1913551

[16] Rola R, Zou Y, Huang TT, Fishman K, Baure J, et al.. (2007) Lack of extracellular superoxide dismutase (EC-SOD) in the microenvironment impacts radiation-induced changes in neurogenesis. Free Radic Biol Med 42: 1133–1145; discussion 1131–1132. 10.1016/j.freeradbiomed.2007.01.020 PMC1934512 17382195

[17] Noble M, Mayer-Proschel M, Proschel C (2005) Redox regulation of precursor cell function: insights and paradoxes. Antioxid Redox Signal 7: 1456–1467. 16356108 10.1089/ars.2005.7.1456

[18] Prozorovski T, Schulze-Topphoff U, Glumm R, Baumgart J, Schroter F, et al (2008) Sirt1 contributes critically to the redox-dependent fate of neural progenitors. Nat Cell Biol 10: 385–394. 18344989 10.1038/ncb1700

[19] Limoli CL, Rola R, Giedzinski E, Mantha S, Huang TT, et al (2004) Cell-density-dependent regulation of neural precursor cell function. Proc Natl Acad Sci U S A 101: 16052–16057. 15522966 10.1073/pnas.0407065101 PMC528770

[20] Tsatmali M, Walcott EC, Crossin KL (2005) Newborn neurons acquire high levels of reactive oxygen species and increased mitochondrial proteins upon differentiation from progenitors. Brain Res 1040: 137–150. 15804435 10.1016/j.brainres.2005.01.087

[21] Tsatmali M, Walcott EC, Makarenkova H, Crossin KL (2006) Reactive oxygen species modulate the differentiation of neurons in clonal cortical cultures. Mol Cell Neurosci 33: 345–357. 17000118 10.1016/j.mcn.2006.08.005 PMC1797198

[22] Maalouf M, Rho JM (2008) Oxidative impairment of hippocampal long-term potentiation involves activation of protein phosphatase 2A and is prevented by ketone bodies. J Neurosci Res 86: 3322–3330. 18646208 10.1002/jnr.21782 PMC2575137

[23] Kamsler A, Segal M (2003) Hydrogen peroxide modulation of synaptic plasticity. J Neurosci 23: 269–276. 12514224 10.1523/JNEUROSCI.23-01-00269.2003 PMC6742148

[24] Knapp LT, Klann E (2002) Role of reactive oxygen species in hippocampal long-term potentiation: contributory or inhibitory? J Neurosci Res 70: 1–7. 12237859 10.1002/jnr.10371

[25] Guo G, Yan-Sanders Y, Lyn-Cook BD, Wang T, Tamae D, et al (2003) Manganese superoxide dismutase-mediated gene expression in radiation-induced adaptive responses. Mol Cell Biol 23: 2362–2378. 12640121 10.1128/MCB.23.7.2362-2378.2003 PMC150726

[26] Fan M, Ahmed KM, Coleman MC, Spitz DR, Li JJ (2007) Nuclear factor-kappaB and manganese superoxide dismutase mediate adaptive radioresistance in low-dose irradiated mouse skin epithelial cells. Cancer Res 67: 3220–3228. 17409430 10.1158/0008-5472.CAN-06-2728

[27] Carpenter M, Epperly MW, Agarwal A, Nie S, Hricisak L, et al (2005) Inhalation delivery of manganese superoxide dismutase-plasmid/liposomes protects the murine lung from irradiation damage. Gene Ther 12: 685–693. 15750616 10.1038/sj.gt.3302468

[28] Epperly M, Bray J, Kraeger S, Zwacka R, Engelhardt J, et al (1998) Prevention of late effects of irradiation lung damage by manganese superoxide dismutase gene therapy. Gene Ther 5: 196–208. 9578839 10.1038/sj.gt.3300580

[29] Epperly MW, Defilippi S, Sikora C, Gretton J, Kalend A, et al (2000) Intratracheal injection of manganese superoxide dismutase (MnSOD) plasmid/liposomes protects normal lung but not orthotopic tumors from irradiation. Gene Ther 7: 1011–1018. 10871749 10.1038/sj.gt.3301207

[30] Greenberger JS, Epperly M, Luketich J, Gooding W, Belani CP (2000) Manganese superoxide dismutase-plasmid/liposome (MnSOD-PL) gene therapy protection of the esophagus from chemoradiotherapy damage during treatment of locally unresectable non-small-cell lung cancer (NSCLC). Clin Lung Cancer 1: 302–304. 14733636 10.3816/clc.2000.n.013

[31] Gauter-Fleckenstein B, Fleckenstein K, Owzar K, Jiang C, Batinic-Haberle I, et al (2008) Comparison of two Mn porphyrin-based mimics of superoxide dismutase in pulmonary radioprotection. Free Radic Biol Med 44: 982–989. 18082148 10.1016/j.freeradbiomed.2007.10.058 PMC3684016

[32] Gauter-Fleckenstein B, Fleckenstein K, Owzar K, Jiang C, Reboucas JS, et al (2010) Early and late administration of MnTE-2-PyP5+ in mitigation and treatment of radiation-induced lung damage. Free Radic Biol Med 48: 1034–1043. 20096348 10.1016/j.freeradbiomed.2010.01.020 PMC3704177

[33] Epperly MW, Smith T, Zhang X, Goff JP, Franicola D, et al (2011) Modulation of in utero total body irradiation induced newborn mouse growth retardation by maternal manganese superoxide dismutase-plasmid liposome (MnSOD-PL) gene therapy. Gene Ther 18: 579–583. 21248791 10.1038/gt.2010.178 PMC3111807

[34] Epperly MW, Wang H, Jones JA, Dixon T, Montesinos CA, et al (2011) Antioxidant-chemoprevention diet ameliorates late effects of total-body irradiation and supplements radioprotection by MnSOD-plasmid liposome administration. Radiat Res 175: 759–765. 21466381 10.1667/RR2398.1 PMC3119545

[35] Guo H, Seixas-Silva JA Jr, Epperly MW, Gretton JE, Shin DM, et al (2003) Prevention of radiation-induced oral cavity mucositis by plasmid/liposome delivery of the human manganese superoxide dismutase (SOD2) transgene. Radiat Res 159: 361–370. 12600239 10.1667/0033-7587(2003)159[0361:porioc]2.0.co;2

[36] Guo HL, Wolfe D, Epperly MW, Huang S, Liu K, et al.. (2003) Gene transfer of human manganese superoxide dismutase protects small intestinal villi from radiation injury. J Gastrointest Surg 7: 229–235; discussion 235–226. 10.1016/s1091-255x(02)00186-5 12600447

[37] Li Y, Huang TT, Carlson EJ, Melov S, Ursell PC, et al (1995) Dilated cardiomyopathy and neonatal lethality in mutant mice lacking manganese superoxide dismutase. Nat Genet 11: 376–381. 7493016 10.1038/ng1295-376

[38] Huang TT, Carlson EJ, Kozy HM, Mantha S, Goodman SI, et al (2001) Genetic modification of prenatal lethality and dilated cardiomyopathy in Mn superoxide dismutase mutant mice. Free Radic Biol Med 31: 1101–1110. 11677043 10.1016/s0891-5849(01)00694-3

[39] Huang TT, Naeemuddin M, Elchuri S, Yamaguchi M, Kozy HM, et al (2006) Genetic modifiers of the phenotype of mice deficient in mitochondrial superoxide dismutase. Hum Mol Genet 15: 1187–1194. 16497723 10.1093/hmg/ddl034

[40] Kim A, Joseph S, Khan A, Epstein CJ, Sobel R, et al (2010) Enhanced expression of mitochondrial superoxide dismutase leads to prolonged in vivo cell cycle progression and up-regulation of mitochondrial thioredoxin. Free Radic Biol Med 48: 1501–1512. 20188820 10.1016/j.freeradbiomed.2010.02.028 PMC2945707

[41] Kim A, Zhong W, Oberley TD (2004) Reversible modulation of cell cycle kinetics in NIH/3T3 mouse fibroblasts by inducible overexpression of mitochondrial manganese superoxide dismutase. Antioxid Redox Signal 6: 489–500. 15130276 10.1089/152308604773934251

[42] Sarsour EH, Venkataraman S, Kalen AL, Oberley LW, Goswami PC (2008) Manganese superoxide dismutase activity regulates transitions between quiescent and proliferative growth. Aging Cell 7: 405–417. 18331617 10.1111/j.1474-9726.2008.00384.x PMC2538945

[43] Quiros-Gonzalez I, Sainz RM, Hevia D, Mayo JC (2011) MnSOD drives neuroendocrine differentiation, androgen independence, and cell survival in prostate cancer cells. Free Radic Biol Med 50: 525–536. 21056653 10.1016/j.freeradbiomed.2010.10.715

[44] Kalen AL, Sarsour EH, Venkataraman S, Goswami PC (2006) Mn-superoxide dismutase overexpression enhances G2 accumulation and radioresistance in human oral squamous carcinoma cells. Antioxid Redox Signal 8: 1273–1281. 16910775 10.1089/ars.2006.8.1273

[45] Fishman K, Baure J, Zou Y, Huang TT, Andres-Mach M, et al (2009) Radiation-induced reductions in neurogenesis are ameliorated in mice deficient in CuZnSOD or MnSOD. Free Radic Biol Med 47: 1459–1467. 19703553 10.1016/j.freeradbiomed.2009.08.016 PMC2767469

[46] West MJ (1999) Stereological methods for estimating the total number of neurons and synapses: issues of precision and bias. Trends Neurosci 22: 51–61. 10092043 10.1016/s0166-2236(98)01362-9

[47] Medeiros DM (2008) Assessing mitochondria biogenesis. Methods 46: 288–294. 18929661 10.1016/j.ymeth.2008.09.026

[48] Tell G, Quadrifoglio F, Tiribelli C, Kelley MR (2009) The many functions of APE1/Ref-1: not only a DNA repair enzyme. Antioxid Redox Signal 11: 601–620. 18976116 10.1089/ars.2008.2194 PMC2811080

[49] Raber J, Villasana L, Rosenberg J, Zou Y, Huang TT, et al (2011) Irradiation enhances hippocampus-dependent cognition in mice deficient in extracellular superoxide dismutase. Hippocampus 21: 72–80. 20020436 10.1002/hipo.20724 PMC2891276

[50] Alamed J, Wilcock DM, Diamond DM, Gordon MN, Morgan D (2006) Two-day radial-arm water maze learning and memory task; robust resolution of amyloid-related memory deficits in transgenic mice. Nat Protoc 1: 1671–1679. 17487150 10.1038/nprot.2006.275

[51] Villeda SA, Luo J, Mosher KI, Zou B, Britschgi M, et al (2011) The ageing systemic milieu negatively regulates neurogenesis and cognitive function. Nature 477: 90–94. 21886162 10.1038/nature10357 PMC3170097

[52] Naylor AS, Bull C, Nilsson MK, Zhu C, Bjork-Eriksson T, et al (2008) Voluntary running rescues adult hippocampal neurogenesis after irradiation of the young mouse brain. Proc Natl Acad Sci U S A 105: 14632–14637. 18765809 10.1073/pnas.0711128105 PMC2567198

[53] van Praag H, Kempermann G, Gage FH (1999) Running increases cell proliferation and neurogenesis in the adult mouse dentate gyrus. Nat Neurosci 2: 266–270. 10195220 10.1038/6368

[54] Fabel K, Wolf SA, Ehninger D, Babu H, Leal-Galicia P, et al (2009) Additive effects of physical exercise and environmental enrichment on adult hippocampal neurogenesis in mice. Front Neurosci 3: 50. 20582277 10.3389/neuro.22.002.2009 PMC2858601

[55] Ehninger D, Kempermann G (2006) Paradoxical effects of learning the Morris water maze on adult hippocampal neurogenesis in mice may be explained by a combination of stress and physical activity. Genes Brain Behav 5: 29–39. 16436186 10.1111/j.1601-183X.2005.00129.x

[56] Rola R, Raber J, Rizk A, Otsuka S, VandenBerg SR, et al (2004) Radiation-induced impairment of hippocampal neurogenesis is associated with cognitive deficits in young mice. Exp Neurol 188: 316–330. 15246832 10.1016/j.expneurol.2004.05.005

[57] Raber J, Rola R, LeFevour A, Morhardt D, Curley J, et al (2004) Radiation-induced cognitive impairments are associated with changes in indicators of hippocampal neurogenesis. Radiat Res 162: 39–47. 15222778 10.1667/rr3206

[58] Rosi S, Ferguson R, Fishman K, Allen A, Raber J, et al (2012) The polyamine inhibitor alpha-difluoromethylornithine modulates hippocampus-dependent function after single and combined injuries. PLoS One 7: e31094. 22299052 10.1371/journal.pone.0031094 PMC3267765

[59] Jaholkowski P, Kiryk A, Jedynak P, Ben Abdallah NM, Knapska E, et al (2009) New hippocampal neurons are not obligatory for memory formation; cyclin D2 knockout mice with no adult brain neurogenesis show learning. Learn Mem 16: 439–451. 19553382 10.1101/lm.1459709

[60] Saxe MD, Malleret G, Vronskaya S, Mendez I, Garcia AD, et al (2007) Paradoxical influence of hippocampal neurogenesis on working memory. Proc Natl Acad Sci U S A 104: 4642–4646. 17360577 10.1073/pnas.0611718104 PMC1810330

[61] Hof PR, Morrison JH (2004) The aging brain: morphomolecular senescence of cortical circuits. Trends Neurosci 27: 607–613. 15374672 10.1016/j.tins.2004.07.013

[62] Bhatt DH, Zhang S, Gan WB (2009) Dendritic spine dynamics. Annu Rev Physiol 71: 261–282. 19575680 10.1146/annurev.physiol.010908.163140

[63] Yang G, Pan F, Gan WB (2009) Stably maintained dendritic spines are associated with lifelong memories. Nature 462: 920–924. 19946265 10.1038/nature08577 PMC4724802

[64] Perez-Cruz C, Nolte MW, van Gaalen MM, Rustay NR, Termont A, et al (2011) Reduced spine density in specific regions of CA1 pyramidal neurons in two transgenic mouse models of Alzheimer’s disease. J Neurosci 31: 3926–3934. 21389247 10.1523/JNEUROSCI.6142-10.2011 PMC6622797

[65] Middei S, Roberto A, Berretta N, Panico MB, Lista S, et al (2010) Learning discloses abnormal structural and functional plasticity at hippocampal synapses in the APP23 mouse model of Alzheimer’s disease. Learn Mem 17: 236–240. 20404004 10.1101/lm.1748310

[66] Caito SW, Milatovic D, Hill KE, Aschner M, Burk RF, et al (2011) Progression of neurodegeneration and morphologic changes in the brains of juvenile mice with selenoprotein P deleted. Brain Res 1398: 1–12. 21636077 10.1016/j.brainres.2011.04.046 PMC3114300

[67] Hood JE, Jenkins JW, Milatovic D, Rongzhu L, Aschner M (2010) Mefloquine induces oxidative stress and neurodegeneration in primary rat cortical neurons. Neurotoxicology 31: 518–523. 20562019 10.1016/j.neuro.2010.05.005

[68] Zaja-Milatovic S, Gupta RC, Aschner M, Montine TJ, Milatovic D (2008) Pharmacologic suppression of oxidative damage and dendritic degeneration following kainic acid-induced excitotoxicity in mouse cerebrum. Neurotoxicology 29: 621–627. 18556069 10.1016/j.neuro.2008.04.009 PMC2517174

[69] Baloyannis SJ, Kim SU (1979) Experimental modification of cerebellar development in tissue culture: x-irradiation induces granular degeneration and unattached purkinje cell dendritic spines. Neurosci Lett 12: 283–288. 460723 10.1016/0304-3940(79)96076-2

[70] Chakraborti A, Allen A, Allen B, Rosi S, Fike JR (2012) Cranial irradiation alters dendritic spine density and morphology in the hippocampus. PLoS One 7: e40844. 22815839 10.1371/journal.pone.0040844 PMC3397939

[71] Hansen KF, Sakamoto K, Wayman GA, Impey S, Obrietan K (2010) Transgenic miR132 alters neuronal spine density and impairs novel object recognition memory. PLoS One 5: e15497. 21124738 10.1371/journal.pone.0015497 PMC2993964

[72] Impey S, Davare M, Lesiak A, Fortin D, Ando H, et al (2010) An activity-induced microRNA controls dendritic spine formation by regulating Rac1-PAK signaling. Mol Cell Neurosci 43: 146–156. 19850129 10.1016/j.mcn.2009.10.005 PMC2818337

[73] Akaneya Y, Sohya K, Kitamura A, Kimura F, Washburn C, et al (2010) Ephrin-A5 and EphA5 interaction induces synaptogenesis during early hippocampal development. PLoS One 5: e12486. 20824214 10.1371/journal.pone.0012486 PMC2930854

[74] Suzuki S, Zhou H, Neumaier JF, Pham TA (2007) Opposing functions of CREB and MKK1 synergistically regulate the geometry of dendritic spines in visual cortex. J Comp Neurol 503: 605–617. 17559089 10.1002/cne.21424

[75] Li Z, Okamoto K, Hayashi Y, Sheng M (2004) The importance of dendritic mitochondria in the morphogenesis and plasticity of spines and synapses. Cell 119: 873–887. 15607982 10.1016/j.cell.2004.11.003

[76] Cuesto G, Enriquez-Barreto L, Carames C, Cantarero M, Gasull X, et al (2011) Phosphoinositide-3-kinase activation controls synaptogenesis and spinogenesis in hippocampal neurons. J Neurosci 31: 2721–2733. 21414895 10.1523/JNEUROSCI.4477-10.2011 PMC6623769

[77] Tsai SY, Hayashi T, Harvey BK, Wang Y, Wu WW, et al (2009) Sigma-1 receptors regulate hippocampal dendritic spine formation via a free radical-sensitive mechanism involving Rac1xGTP pathway. Proc Natl Acad Sci U S A 106: 22468–22473. 20018732 10.1073/pnas.0909089106 PMC2792161

[78] Quijano C, Romero N, Radi R (2005) Tyrosine nitration by superoxide and nitric oxide fluxes in biological systems: modeling the impact of superoxide dismutase and nitric oxide diffusion. Free Radic Biol Med 39: 728–741. 16109303 10.1016/j.freeradbiomed.2005.04.014

[79] Hawkins RD, Son H, Arancio O (1998) Nitric oxide as a retrograde messenger during long-term potentiation in hippocampus. Prog Brain Res 118: 155–172. 9932440 10.1016/s0079-6123(08)63206-9

[80] Paul V, Ekambaram P (2011) Involvement of nitric oxide in learning & memory processes. Indian J Med Res 133: 471–478. 21623030 PMC3121276

[81] Kasischke KA, Vishwasrao HD, Fisher PJ, Zipfel WR, Webb WW (2004) Neural activity triggers neuronal oxidative metabolism followed by astrocytic glycolysis. Science 305: 99–103. 15232110 10.1126/science.1096485

